# Comparison of corneal aberrations from anterior segment swept source OCT versus Placido-topography combined spectral domain OCT in cataract patients

**DOI:** 10.1186/s40662-023-00348-z

**Published:** 2023-08-01

**Authors:** Stefan Georgiev, Manuel Ruiss, Andreea Dana-Fisus, Rainer A. Leitgeb, Oliver Findl

**Affiliations:** 1 VIROS-Vienna Institute for Research in Ocular Surgery, A Karl Landsteiner Institute, Hanusch Hospital, Heinrich-Collin-Strasse 30, 1140 Vienna, Austria; 2 Center for Medical Physics and Biomedical Engineering, Vienna, Austria

**Keywords:** Corneal aberrations, Wavefront analysis, Optical coherence tomography, Cataract surgery

## Abstract

**Background:**

To comprehensively evaluate the agreement of component corneal aberrations from the newly updated wavefront analysis software of a swept-source optical coherence tomographer (SS-OCT) and a referential Placido-topography combined OCT device in elderly cataract patients.

**Methods:**

Retrospective study including 103 eyes from 103 elderly patients scheduled for cataract surgery that were measured on the same day with a SS-OCT (Heidelberg Engineering, Germany) device and a Placido-topography combined OCT device (CSO, Italy). Anterior, total, and posterior corneal wavefront aberrations were evaluated for their mean differences and limits of agreement (LoA) via Bland-Altman plots. Vector analysis was additionally employed to compare corneal astigmatism measurements in dioptric vector space.

**Results:**

Mean differences of all corneal aberrometric parameters did not exceed 0.05 μm. Total corneal aberrations were not significantly different from 0 except for vertical coma (− 0.04 μm; *P* = 0.003), spherical aberration (− 0.01 μm, *P* < 0.001), and root mean square (RMS) higher-order aberration (HOA) (0.03 μm, *P* = 0.04). The 95% LoA for total corneal aberration parameters between both devices were − 0.46 to 0.42 μm for horizontal astigmatism, − 0.37 to 0.41 μm for oblique astigmatism, − 0.19 to 0.17 μm for oblique trefoil, − 0.33 to 0.25 μm for vertical coma, − 0.20 to 0.22 μm for horizontal coma, − 0.22 to 0.20 μm for horizontal trefoil, − 0.11 to 0.08 μm for spherical aberration, and − 0.22 to 0.28 μm for RMS HOA. Vector analysis revealed no statistically significant mean differences for anterior, total, and posterior corneal astigmatism in dioptric vector space.

**Conclusion:**

In eyes undergoing cataract surgery with a regular elderly cornea, corneal wavefront analysis from the SS-OCT device showed functional equivalency to the reference device. Nevertheless, clinically relevant higher order aberration parameters should be interpreted with caution for surgical decision-making.

## Background

High-resolution cross-sectional imaging has become crucial in clinical ophthalmology to analyze ocular structures of the anterior segment for various applications [1]. To adequately characterize the spatial correspondence between anterior and posterior corneal surfaces is also of increasing interest in refractive cataract surgery [2]. Placido topography-based reflective imaging is well-established for anterior corneal surface analysis, and frequently complements imaging modalities that generate 3-dimentional corneal elevation data. This is because the former acquires the image of the pre-corneal tear film in a single shot with well-documented accuracy and resolution, which can be interlaced with tomographic cross-sectional images that are subject to longer acquisition times [3]. On the other hand, Fourier-domain optical coherence tomography (OCT) has significantly improved high-resolution segmentation and imaging speed, which has paved the way for spectral-domain OCT (SD-OCT) and swept-source OCT (SS-OCT) to be employed for full 3-dimentional biometry in anterior segment surgery [4, 5]. The latter, in particular, with its high reproducibility and longer center wavelength for enhanced penetration in dense cataracts, may at present be poised to become the gold standard for this application [1, 6].

At the turn of the millennium, the advent of ocular wavefront analysis has also ushered in a paradigmatic shift on how clinicians are able to understand contributions and intricacies of ocular aberrations on visual quality [7]. Corneal elevation data can be analogously described via orthonormal polynomials from the Zernike expansion to additionally characterize constituent monochromatic higher-order aberrations (HOAs) of the wavefront error function [8, 9]. Its application has been mainly confined to assessments of corneal irregularities in corneal refractive surgery, diagnosis and management of corneal ectatic diseases, and orthokeratology [10].

Recent improvements in cataract surgery, however, have been accompanied by a frenetic pace at which increasingly diverse intraocular lens (IOL) designs have been made available [11]. This has effectively blurred the line between cataract and refractive surgery, with an abundant selection of IOLs that can not only correct refractive errors along with pre-existing corneal astigmatism or spherical aberration (SA), but also alleviate presbyopia by inducing multifocality or extending the depth of focus [12–14]. With corneal aberrations being coupled to the IOL design of choice to form the retinal image, their characterization will be of increasing importance for patient counseling and informed surgical decision-making.

A recently introduced anterior segment SS-OCT ANTERION (Heidelberg Engineering, Germany) is a multimodal platform which solely uses a tunable long wavelength swept-source laser for imaging [1]. Its ability to measure biometric parameters has been validated with equal or superior repeatability in comparison with different devices—although some values have not shown consistent interchangeability [15–18]. Nonetheless, since its former software versions (until version 1.3.) only enabled the export of Zernike polynomials up to one decimal place, the device has yet to be evaluated for its ability to optically characterize corneal shape irregularities.

With its latest software upgrade solving this issue (version 1.4.), the aim of this study was to evaluate the interchangeability of component corneal aberrations measured by the SS-OCT platform with a referential Placido-topography combined SD-OCT device in cataract patients.

## Methods

### Subjects

This retrospective study included patients scheduled for routine cataract surgery between January 2020 and November 2022 that were preoperatively sequentially measured with the ANTERION SS-OCT (Heidelberg Engineering, Germany) and the MS-39 Placido + SD-OCT (CSO, Italy) once on the same day by three different experienced observers. Only healthy eyes (except non-fixation threatening cataract) were included. Exclusion criteria were corneal pathologies (i.e., keratoconus), previous refractive and cataract surgery, any diseases or conditions that would prevent adequate fixation (including dense cataracts), or other ophthalmic diseases and conditions that could have an impact on the tear-film or corneal integrity (i.e., dry eye disease, trauma, or corneal scars). Patients were instructed to blink twice before each measurement to avoid tear film break-up, and only measurements that fulfilled the quality control parameters of both devices were considered for analysis.

#### Measurement devices

The ANTERION SS-OCT uses a swept-source laser at a center wavelength of 1300 nm to generate anterior segment images with in-tissue axial and transversal resolutions of < 10 and < 45 µm, respectively [1]. Its long-wavelength swept-source laser with reduced sensitivity roll-off acquires non-invasively 50,000 A-scans to compose a dense wide-reaching scan pattern for the entire anterior segment and posterior lens. Using an active eye-tracker, tomographic cross-sectional images model the cornea 3-dimensionally at an 8 mm diameter in less than a second with a radial scan pattern consisting of 65 radial B-Scans; each of them comprised of 256 A-Scan lines. In addition to computing conventional maps for both corneal surfaces (curvature, elevation, pachymetry, and dioptric power of both corneal surfaces) the manufacturers’ investigational software has now been updated (version 1.4.2.0.) to quantify elevation data corneal wavefront aberrations to the second decimal place. This is automatically generated via ray-tracing for the anterior corneal surface in addition to the total cornea by incorporating both corneal surfaces together with the aqueous refractive index.

The MS-39 is an anterior segment SD-OCT imaging and biometry platform, and like its Scheimpflug-based predecessor, is combined with Placido topography. The device acquires non-invasively anterior segment images at a center wavelength of 840 nm with in-tissue axial and transversal resolutions of 3.5 µm and 35 µm, respectively. In our outpatient clinic, 3-dimentional tomographic modelling of the cornea was always generated according to a standard protocol: 25 radial SD-OCT scans each comprised of 1024 A-scan lines measured both corneal surfaces together with the corneal thickness, which was interlaced with the Placido-based anterior corneal image via the manufacturers’ proprietary algorithm. In addition to conventional corneal maps (elevation, curvature, pachymetry, and dioptric power of both corneal surfaces), 2nd to 7th order Zernike polynomials (excluding defocus) are automatically computed via ray-tracing for anterior, total, and posterior corneal contributions (Phoenix software version 4.0).

#### Statistical analysis

In accordance with the ANSI standard (Z80.28), the corneal wavefront error was referenced with each of the devices’ software to the pupil center, since it is the light that passes and leaves through the entrance and exit pupil that forms the retinal image [19]. We opted to compute aberrations for a 5-mm pupil, because this zone has been frequently employed in comprehensive component ocular aberrations studies [20–22], and patients of various age groups implanted with presbyopia-correcting IOLs have shown to have a mesopic pupil size of 5 mm or less [23].

From both SS-OCT and Placido + SD-OCT, anonymized datasets of anterior and total corneal Zernike coefficients were directly exportable as.csv files on Excel (version 16.67, Microsoft Corp.). The respective posterior corneal aberration coefficients were then obtained via direct subtraction (total aberration coefficients − anterior aberration coefficients). The following parameters were common to both devices: oblique astigmatism, with-the-rule (WTR)/against-the-rule (ATR) astigmatism, oblique trefoil, horizontal trefoil, vertical coma, horizontal coma, SA, and root mean square (RMS) HOA computed from the 3rd to 6th orders.

Since aberrations between eyes are correlated, only one eye from each patient was randomly selected for statistical analysis. To account for corneal enantiomorphism, coefficients affected by midline symmetry that would otherwise statistically cancel each other out were transposed from left to right eyes (i.e., Z(2, − 2), Z(3, + 1), and Z(3, + 3)) [24]. Further, for a more clinically intuitive comparison, Z(2, + 2) horizontal WTR/ATR astigmatism and Z(2, − 2) oblique astigmatism were also converted via Fourier transformation into power vector form J0 and J45 [25]. A positive J0 value indicates WTR astigmatism, a negative value ATR astigmatism. A positive J45 value represents oblique astigmatism whose power is greatest at 135°, and conversely, a negative J45 value represents the astigmatic power being greatest at 45°.

Statistical analysis was conducted with IBM SPSS Statistics for MAC (software version 27.0, IBM Corp.) Descriptive statistics were employed for mean values with corresponding standard deviations (SDs) for anterior, total, and posterior corneal aberrations. The normality of data distributions was tested with the Kolmogorov-Smirnov test, and parametric statistics for testing the hypothesis were only used if the assumption of a normal distribution was met. For each of the measured parameters, the paired t-test or the Wilcoxon signed rank test were employed to determine the statistical significance of the mean difference. A *P* value of < 0.05 was considered statistically significant. Mean differences and 95% limits of agreement (LoA) were assessed via Bland-Altman plots by plotting inter-device differences against their average [26].

## Results

A hundred and three eyes (right eye 52, left eye 51) of 103 patients (men 52, women 51) measured with both devices from January 2020 to November 2022 that met the inclusion criteria were evaluated. The mean patients’ age with corresponding SD was 68 ± 9 years (range: 38 to 85 years).

### Comparison of measurements

Zernike coefficients from both devices had overall similar mean and mean absolute values, with matching sign orientations and corresponding SDs for the anterior, total, and posterior cornea as summarized in Table 1. The mean values for anterior and total corneal parameters were not statistically significant except for minor differences for anterior and total vertical coma (*P* = 0.002; *P* = 0.01), anterior and total SA (*P* < 0.001; *P* = 0.006), and total RMS HOA (*P* = 0.04). For the posterior cornea, the main coefficient that altered the total corneal aberration profile was WTR/ATR astigmatism from both devices (mean value: 0.18 μm, SS-OCT; 0.16 μm, Placido + SD-OCT). With posterior corneal parameters having otherwise substantially lower magnitudes, minor differences were nevertheless observable for posterior WTR/ATR astigmatism (*P* < 0.001), posterior vertical coma (*P* < 0.001), posterior SA (*P* < 0.001), and posterior HOA RMS (*P* = 0.03).

**Table 1:** Comparison of corneal aberration measurements between SS-OCT and Placido + SD-OCT (5 mm zone; pupil centered)

Parameter	Mean ± SD (absolute mean)		Range		P*
	SS-OCT	Placido + SD-OCT	SS-OCT	Placido + SD-OCT	
Anterior cornea (μm)					
Z(2, − 2) Oblique astigmatism	0.14 ± 0.28 (0.23)	0.16 ± 0.27 (0.24)	− 0.48, + 1.06	− 0.44, + 0.98	0.16
Z(2, + 2) WTR/ATR astigmatism	− 0.02 ± 0.50 (0.38)	− 0.02 ± 0.47 (0.36)	− 1.26, + 1.25	− 1.20, + 1.17	0.71
Z(3, − 3) Oblique trefoil	− 0.10 ± 0.10 (0.12)	− 0.10 ± 0.12 (0.13)	− 0.34, + 0.16	− 0.47, + 0.24	0.53
Z(3, − 1) Vertical coma	− 0.01 ± 0.15 (0.11)	− 0.06 ± 0.17 (0.13)	− 0.57, + 0.56	− 0.87, + 0.45	**0.002**
Z(3, + 1) Horizontal coma	− 0.15 ± 0.13 (0.17)	− 0.14 ± 0.13 (0.16)	− 0.58, + 0.29	− 0.47, + 0.34	0.37
Z(3, + 3) Horizontal trefoil	0.02 ± 0.10 (0.08)	0.02 ± 0.11 (0.08)	− 0.24, + 0.26	− 0.32, + 0.32	0.55
Z(4,0) Spherical aberration	0.17 ± 0.07 (0.17)	0.15 ± 0.07 (0.15)	0.00, + 0.56	− 0.02, + 0.57	** < 0.001**
HOA RMS	0.36 ± 0.10 (0.36)	0.37 ± 0.14 (0.37)	+ 0.13, + 0.69	+ 0.19, + 1.01	0.43†
Total cornea (μm)					
Z(2, − 2) Oblique astigmatism	0.14 ± 0.26 (0.22)	0.16 ± 0.25 (0.23)	− 0.43, + 0.89	− 0.40, + 0.86	0.31
Z(2, + 2) WTR/ATR astigmatism	0.16 ± 0.47 (0.38)	0.13 ± 0.45 (0.36)	− 0.99, + 1.26	− 0.99, + 1.31	0.38
Z(3, − 3) Oblique trefoil	− 0.09 ± 0.10 (0.11)	− 0.09 ± 0.12 (0.12)	− 0.31, + 0.16	− 0.45, + 0.25	0.32
Z(3, − 1) Vertical coma	− 0.03 ± 0.15 (0.11)	− 0.07 ± 0.17 (0.14)	− 0.62, + 0.53	− 0.93, + 0.42	**0.01**
Z(3, + 1) Horizontal coma	− 0.15 ± 0.07 (0.16)	− 0.14 ± 0.12 (0.15)	− 0.55, + 0.25	− 0.47, + 0.31	0.41
Z(3, + 3) Horizontal trefoil	0.02 ± 0.09 (0.08)	0.01 ± 0.13 (0.09)	− 0.24, + 0.23	− 0.70, + 0.30	0.34
Z(4,0) Spherical aberration	0.15 ± 0.07 (0.15)	0.14 ± 0.08 (0.14)	0.00, + 0.53	− 0.08, + 0.55	**0.006**
HOA RMS	0.34 ± 0.11 (0.34)	0.37 ± 0.16 (0.37)	+ 0.12, + 0.73	+ 0.19, + 1.10	**0.04**†
Posterior cornea (μm)					
Z(2, − 2) Oblique astigmatism	0.00 ± 0.05 (0.04)	− 0.01 ± 0.08 (0.04)	− 0.17, + 0.12	− 0.60, + 0.14	0.31†
Z(2, + 2) WTR/ATR astigmatism	0.18 ± 0.08 (0.18)	0.16 ± 0.19 (0.18)	0.01, + 0.39	− 1.59, + 0.53	** < 0.001**†
Z(3, − 3) Oblique trefoil	0.01 ± 0.02 (0.02)	0.01 ± 0.04 (0.03)	− 0.03, + 0.04	− 0.23, + 0.10	0.76†
Z(3, − 1) Vertical coma	− 0.02 ± 0.03 (0.03)	− 0.01 ± 0.03 (0.03)	− 0.12, + 0.07	− 0.19, + 0.12	** < 0.001**†
Z(3, + 1) Horizontal coma	0.01 ± 0.01 (0.01)	0.01 ± 0.02 (0.01)	− 0.04, + 0.04	− 0.03, + 0.10	0.16†
Z(3, + 3) Horizontal trefoil	− 0.01 ± 0.01(0.01)	− 0.01 ± 0.06 (0.02)	− 0.04, + 0.03	− 0.59, + 0.06	0.46†
Z(4, 0) Spherical aberration	− 0.02 ± 0.01(0.02)	− 0.01 ± 0.02 (0.01)	− 0.05, + 0.01	− 0.17, + 0.02	** < 0.001**†
HOA RMS	0.05 ± 0.02 (0.05)	0.06 ± 0.07 (0.06)	+ 0.01, + 0.14	+ 0.01, + 0.66	**0.03**†

*SD-OCT* = spectral-domain optical coherence tomography; *SS-OCT* = swept-source optical coherence tomography; *SD* = standard deviation; *WTR* = with-the-rule; *ATR* = against-the-rule; *HOA* = higher-order aberration; *RMS* = root mean square

*Paired t*-*test, significant at *P* < 0.05

†Wilcoxon signed rank test, significant at *P* < 0.05. *P* values indicated in bold refer to statistically significant results

**Fig. 1:**
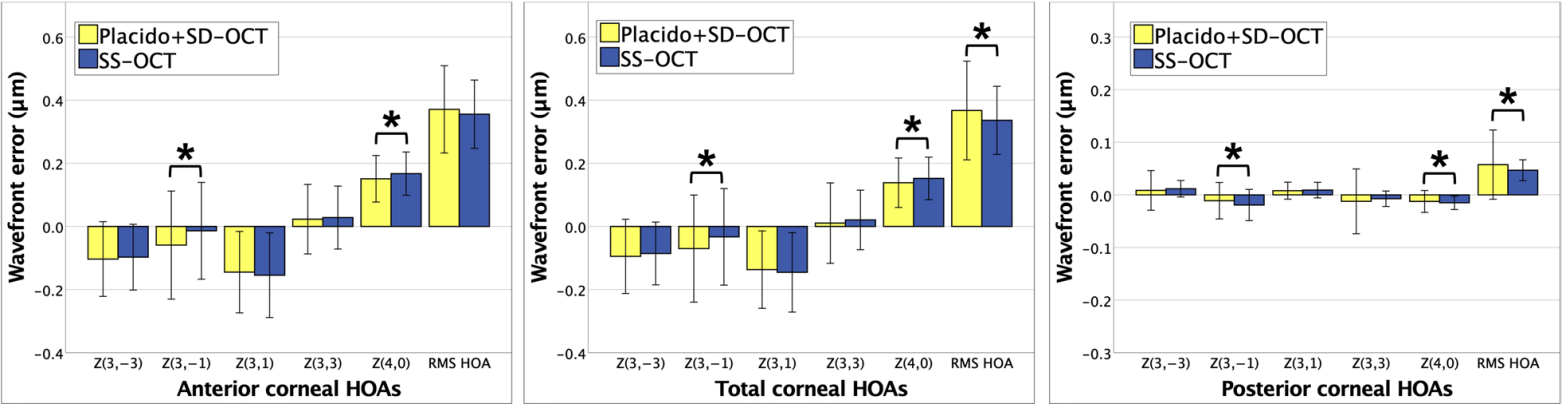
Anterior, total, and posterior corneal higher-order aberrations (HOAs, 5 mm zone, pupil centered). Error bars indicate mean ± SD. RMS, root mean square; Asterisk indicates a statistically significant difference

Figure 1 shows bar-graphs for anterior, total, and posterior corneal HOAs. On average, anterior/total corneal HOAs with a directional orientation at a 5-mm pupil were oblique trefoil (negative), horizontal coma (negative), and SA (positive). Posterior corneal HOAs had means close to 0, and all except vertical coma were of opposite sign to their anterior counterparts—thereby only slightly compensating the anterior corneal wavefront.

### Agreement of measurements

Table 2 shows mean differences, statistical differences from 0, and 95% LoAs of measured corneal aberrations between both devices. Mean differences [(Placido + SD-OCT) − (SS-OCT)] were not significantly different from 0, except for the coefficients that exhibited statistically different inter-device means (see Table 1). Nevertheless, the inter-device bias was overall negligible, with mean differences of all measured parameters not exceeding ± 0.05 μm.

**Table 2:** Inter-device agreement of corneal aberration measurements (5 mm zone; pupil centered)

Parameter	Mean difference ± SD	P*	95% LoA	
			Lower, upper	Range
Anterior cornea (μm)				
Z(2, − 2) Oblique astigmatism	0.03 ± 0.20	0.16	− 0.37, + 0.43	0.80
Z(2, + 2) WTR/ATR astigmatism	0.01 ± 0.29	0.71	− 0.62, + 0.65	1.27
Z(3, − 3) Oblique trefoil	0.00 ± 0.10	0.55	− 0.19, + 0.18	0.37
Z(3, − 1) Vertical coma	− 0.05 ± 0.15	**0.003**	− 0.34, + 0.25	0.59
Z(3, + 1) Horizontal coma	0.01 ± 0.11	0.38	− 0.21, + 0.23	0.44
Z(3, + 3) Horizontal trefoil	0.00 ± 0.09	0.59	− 0.18, + 0.17	0.35
Z(4,0) Spherical aberration	− 0.02 ± 0.05	** < 0.001**	− 0.11, + 0.08	0.19
HOA RMS	0.02 ± 0.10	0.42†	− 0.18, + 0.21	0.39
Total cornea (μm)				
Z(2, − 2) Oblique astigmatism	0.02 ± 0.20	0.16	− 0.37, + 0.41	0.78
Z(2, + 2) WTR/ATR astigmatism	− 0.03 ± 0.20	0.71	− 0.46, + 0.42	0.88
Z(3, − 3) Oblique trefoil	0.00 ± 0.09	0.48	− 0.19, + 0.17	0.36
Z(3, − 1) Vertical coma	− 0.04 ± 0.15	**0.003**	− 0.33, + 0.25	0.58
Z(3, + 1) Horizontal coma	0.01 ± 0.11	0.38	− 0.20, + 0.22	0.42
Z(3, + 3) Horizontal trefoil	− 0.01 ± 0.11	0.59	− 0.22, + 0.20	0.42
Z(4,0) Spherical aberration	− 0.01 ± 0.05	** < 0.001**	− 0.11, + 0.08	0.19
HOA RMS	0.03 ± 0.13	**0.04**†	− 0.22, + 0.28	0.50
Posterior cornea (μm)				
Z(2, − 2) Oblique astigmatism	− 0.01 ± 0.07	0.23†	− 0.14, + 0.12	0.26
Z(2, + 2) WTR/ATR astigmatism	− 0.03 ± 0.20	** < 0.001**†	− 0.42, + 0.36	0.77
Z(3, − 3) Oblique trefoil	0.00 ± 0.04	0.67†	− 0.08, + 0.07	0.15
Z(3, − 1) Vertical coma	0.01 ± 0.02	** < 0.001**†	− 0.03, + 0.05	0.08
Z(3, + 1) Horizontal coma	0.01 ± 0.01	0.13†	− 0.03, + 0.03	0.06
Z(3, + 3) Horizontal trefoil	0.00 ± 0.06	0.49†	− 0.12, + 0.11	0.23
Z(4,0) Spherical aberration	0.01 ± 0.02	** < 0.001**†	− 0.04, + 0.04	0.08
HOA RMS	0.01 ± 0.06	**0.03**†	− 0.10, + 0.12	0.22

*SD-OCT* = spectral-domain optical coherence tomography; *SS-OCT* = swept-source optical coherence tomography; *SD* = standard deviation; *LoA* = limits of agreement; *WTR* = with-the-rule; *ATR* = against-the-rule; *HOA* = higher order aberration; *RMS* = root mean square

*One sided t test, significant at *P* < 0.05

†Wilcoxon signed rank test, significant at *P* < 0.05. *P* values indicated in bold refer to statistically significant results

**Fig. 2:**
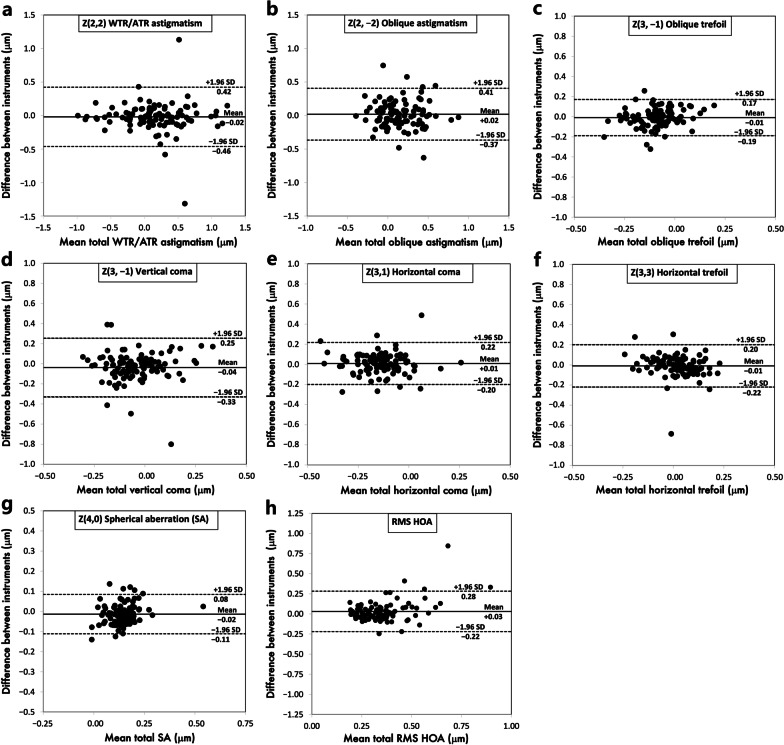
Bland-Altman plots of the agreement between Placido + SD-OCT and SS-OCT for total WTR/ATR astigmatism (**a**), total oblique astigmatism (**b**), total oblique trefoil (**c**), total vertical coma (**d**), total horizontal coma (**e**), total horizontal trefoil (**f**), total SA (**g**), and total RMS HOA (**h**). Solid lines indicate mean difference; dashed outer lines indicate 95% limits of agreement. SD-OCT, spectral-domain optical coherence tomography; SS-OCT, swept-source optical coherence tomography; WTR, with-the-rule; ATR, against-the-rule; SA, spherical aberration; RMS, root mean square; HOA, higher-order aberrations

The 95% LoAs agreement ranges were generally proportional to the magnitudes of individual Zernike coefficients; in detail, the Bland-Altman plots for all measured total corneal aberration parameters are given in Fig. 2.

### Astigmatic vector analysis

**Fig. 3:**
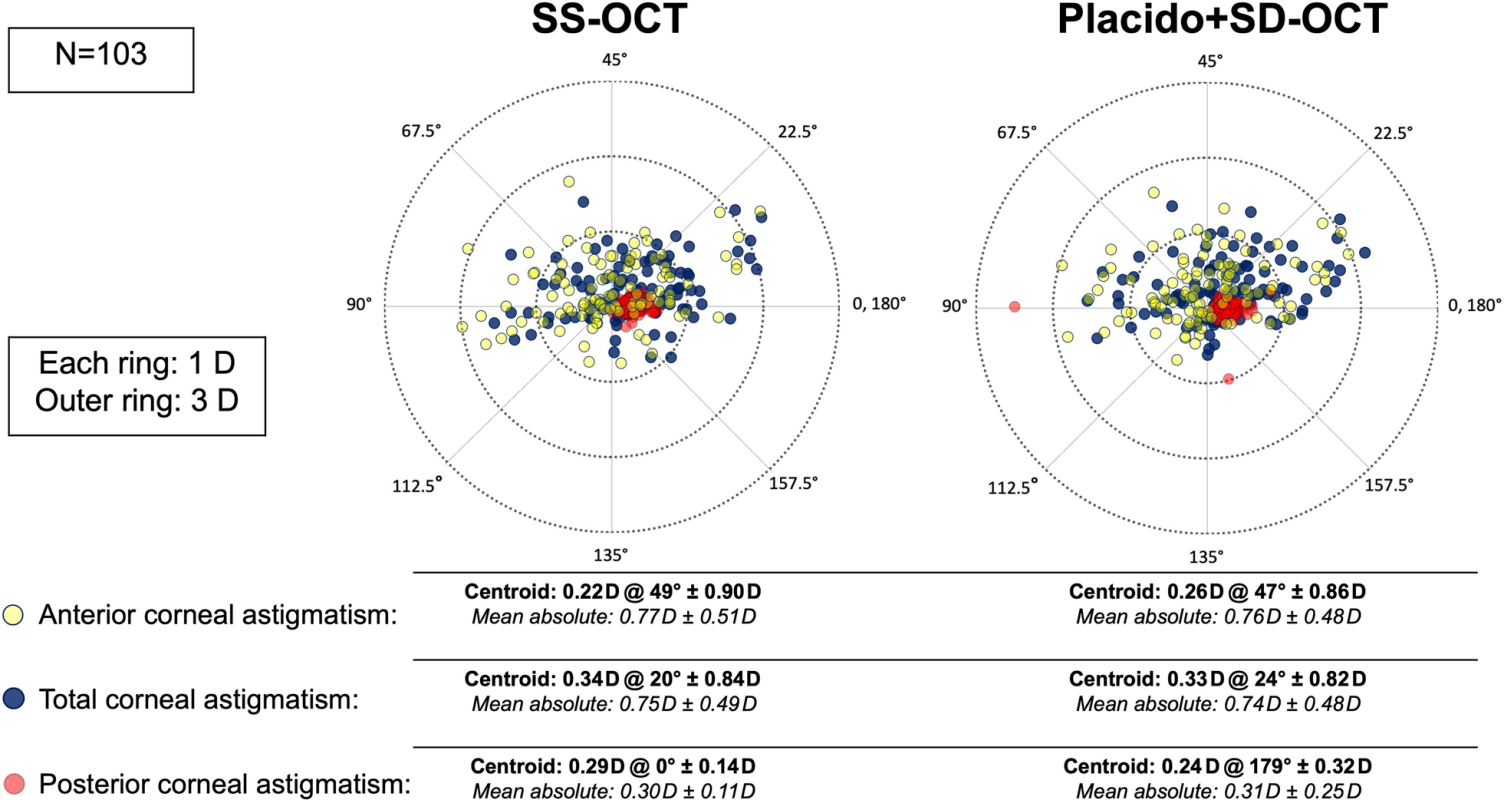
Double-angle plots of corneal astigmatism converted from anterior, total, and posterior astigmatic Zernike coefficients for SS-OCT (left) and Placido + SD-OCT (right). SD-OCT, spectral-domain optical coherence tomography; SS-OCT, swept-source optical coherence tomography

**Fig. 4:**
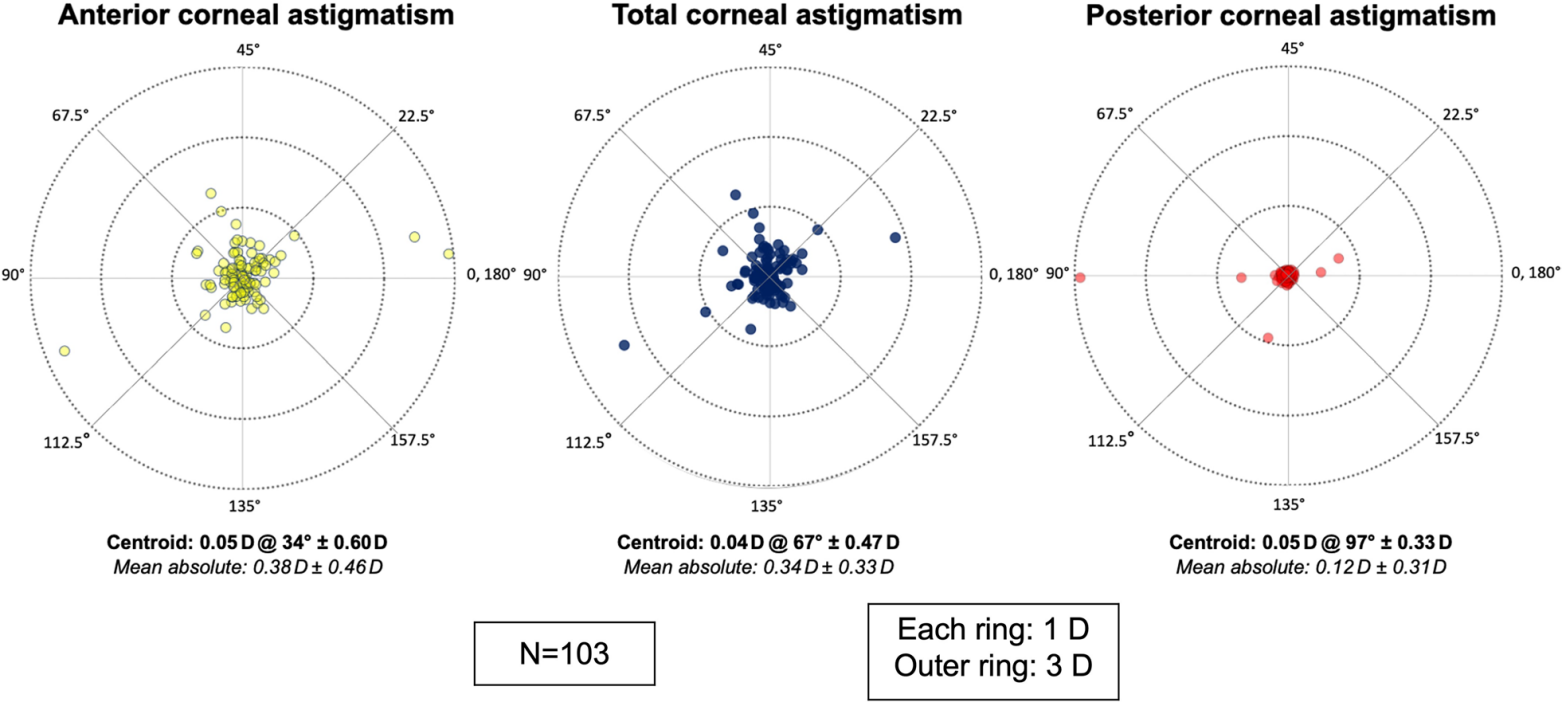
Double-angle plots of the differences in corneal astigmatism between Placido + SD-OCT and SS-OCT [(Placido + SD-OCT) − (SS-OCT)] converted from astigmatic Zernike coefficients. SD-OCT, spectral-domain optical coherence tomography; SS-OCT, swept-source optical coherence tomography

Converted from μm into the dioptric vector space (J0, J45), anterior, total, and posterior corneal astigmatic values had inter-device mean differences of ≤  ± 0.02 D [(Placido + SD-OCT) − (SS-OCT)], with none of them being significantly different as summarized in Table 3. From both devices, anterior corneal J0 values were close to 0, revealing on average an equal proportion of anterior corneal WTR and ATR astigmatism in our population. Total corneal J0 exhibited on average a shift towards WTR astigmatism with more power at 0, 180°, which incorporates the posterior corneal surface as a low-powered negative lens whose steeper curvature in the vertical meridian created ATR astigmatism in most cases. Anterior and total corneal J45 values were of quasi-equal magnitudes for each of the devices, with posterior corneal J45 having a negligible contribution. Figure 3 shows the corresponding double-angle plots converted from astigmatic coefficients of the polynomial expansion, with measurements from both devices having quasi-equivalent centroids and mean absolute cylinder magnitudes. Figure 4 shows the respective inter-device double-angle difference plots [(Placido + SD-OCT) − (SS-OCT)].

**Table 3:** Inter-device agreement of corneal astigmatism in dioptric vector space

Parameter	Mean ± SD		Mean difference ± SD	*P*	95% LoA	
	SS-OCT	Placido + SD-OCT			Lower, upper	Span
Anterior cornea (D)						
J0	− 0.02 ± 0.39	− 0.01 ± 0.38	− 0.01 ± 0.25	0.71	− 0.49, 0.51	1.00
J45	0.11 ± 0.22	0.13 ± 0.21	0.02 ± 0.20	0.31	− 0.37, 0.41	0.78
Total cornea (D)						
J0	0.13 ± 0.39	0.11 ± 0.36	− 0.02 ± 0.18	0.38	− 0.36, 0.33	0.69
J45	0.11 ± 0.20	0.12 ± 0.20	0.02 ± 0.15	0.31	− 0.29, 0.32	0.61
Posterior cornea (D)						
J0	0.14 ± 0.06	0.12 ± 0.15	− 0.02 ± 0.15	0.11	− 0.33, 0.28	0.61
J45	0.00 ± 0.04	0.01 ± 0.06	− 0.01 ± 0.05	0.20	− 0.11, 0.09	0.20

*SD* = standard deviation; *D* = diopter; *LoA* = limits of agreement; *SD-OCT* = spectral-domain optical coherence tomography; *SS-OCT* = swept-source optical coherence tomography

## Discussion

Although there is no established gold standard for corneal aberrometry, numerous reports have evaluated anterior corneal aberrations by fitting Zernike polynomials to Placido-topography height maps [2, 3, 8, 9, 20, 21]. The Placido + SD-OCT device (MS-39) was also validated with different Scheimpflug-based tomographers such as the Pentacam (OCULUS Optikgeräte GmbH) [27], and has exhibited excellent repeatability and high agreement with SS-OCT-based biometry (Argos; Movu, Inc) [28]. More recently, Schiano-Lomoriello et al. have shown that most biometric parameters between Placido + SD-OCT (MS-39) and SS-OCT (ANTERION) can be considered to be clinically interchangeable [16]. Herber et al., however, found the LoAs of most corneal parameters between Scheimpflug imaging (Pentacam HR), dual Placido-combined Scheimpflug imaging (Galilei G6, Ziemer Ophthalmic Systems), and SS-OCT (ANTERION) to be too broad to be eligible for clinical interchangeability [17]. We therefore evaluated the agreement and interchangeability of wavefront aberrations at the refracting components of the cornea between the SS-OCT platform and the referential Placido + SD-OCT device.

In our study, even though statistically significant mean differences between both devices were observable for some of the coefficients, none of them exceeded 0.1 μm, which is generally considered to be a clinically meaningful increment. At a 5 mm zone referenced to the pupil center, the component anterior, total, and posterior corneal coefficients from both devices were also congruent with the comprehensive study by Atchison et al. using the Pentacam [22]: WTR/ATR astigmatism being the most prominent corneal aberration, followed by oblique astigmatism, horizontal coma, and SA—with posterior corneal aberrations other than WTR/ATR astigmatism having a negligible compensatory effect on the anterior corneal surface. Otherwise, anterior corneal aberration magnitudes from both devices were also similar to previous reports that evaluated anterior corneal aberration coefficients at that pupil size from topography (Visser et al. [20]; Philip et al. [21]). The main difference compared to the aforementioned studies that had evaluated healthy younger subjects, were the overall higher mean HOA values in our population [20–22]. This is to be expected, given that the cornea is known to become less symmetrical with age [9]. Interestingly, corneal SA was also observed to be about 0.01–0.03 μm higher in our study. Although the increase in ocular SA has shown to be mainly attributable to a gradual age-related decoupling of corneal and internal surface aberrations [9], this would be in agreement with the study by Sicam et al. where both corneal surfaces exhibited a slight increase with age [29].

We consider astigmatism measurements computed from the polynomial decomposition to be clinically interchangeable (Table 3; Figs. 3, 4), given that the equivalent centroids and high inter-device vector agreements are in accordance with astigmatism agreement from keratometry/topography [2]. The distribution of anterior corneal astigmatism in our cataract patient population was also reflective of the known age-related shift towards ATR astigmatism [30], while posterior corneal astigmatism, as a largely age-independent stable parameter (mean: − 0.30 ± 0.11 D, SS-OCT; − 0.31 ± 0.25 D, Placido + SD-OCT), was congruent with previously published data (Koch et al. [30], mean: − 0.30 ± 0.15 D, dual Scheimpflug imaging).

Total corneal HOA agreement in our study was also similar to a more recent study by Piccinini et al. comparing Scheimpflug imaging (Pentacam) and dual Placido-combined Scheimpflug imaging (Galilei G4) in healthy eyes [31]. In that study, HOAs also showed moderate correlations with no directional inter-device bias for individual Zernike coefficients, albeit with somewhat broader inter-device 95% LoAs than ours. The authors concluded that they were reasonably equivalent for clinical use if values are used with caution for diagnostic or therapeutic purposes. Nevertheless, when it comes to clinical HOA interchangeability, parameters such as corneal SA are of interest for surgical planning and must be judged by their clinical applicability. Aspheric IOLs are designed to compensate at fixed amounts for the usually prolate corneal curvature [13], whereas recently introduced non-diffractive wavefront-shaping IOLs mainly differ in the amount and polarity of induced SA of different orders for extending the depth of focus [32, 33]. While in our study corneal SA was the total HOA coefficient with the highest inter-device agreement (95% LoA range: 0.19 μm, Table 2; Fig. 2), this range would in our opinion still be too broad to be considered interchangeable for appropriating IOL designs to patient-specific corneal asphericity.

On the other hand, HOAs decrease contrast sensitivity and pose a risk for intolerable postoperative photic phenomena such as glare and halo in refractive cataract surgery. This is insofar increasingly relevant, because the line between presbyopia-correcting and monofocal IOLs has been blurred with the emergence of enhanced monofocal IOLs [11]. Corneal RMS HOA can therefore be a useful screening parameter, since IOL designs for enhancing patients’ range of vision necessarily go along with a drop in distance contrast sensitivity. However, notwithstanding some outliers from Placido + SD-OCT that broadened the agreement range (95% LoA range 0.50 μm, Table 2), inter-device variability increased above the 0.4 μm mark (Fig. 2). This would exclude clinical interchangeability because a HOA RMS of > 0.3 μm within a 4-mm pupil has been tentatively postulated as a relative contraindication for multifocal IOL implantation [3]. Although this is not based on empirical data, the reasoning behind this is that a RMS of 0.29 μm is equal to a 0.5 D defocus blur for a 4-mm pupil (rescaled to 0.45 μm for a 5-mm pupil). In our study, 18 (17%) and 14 (14%) out of 103 eyes measured by Placido + SD-OCT and SS-OCT would have fallen into that category, respectively. This exemplifies how the impact of HOAs on corneal optical quality is still underexplored to quantitatively derive cut-off values for various IOL platforms, which is compounded by the fact that for presbyopia-correcting IOLs, there is a paucity of data as to how much contrast sensitivity deterioration would be tolerable for patients with (or at risk for) ocular comorbidities [34].

Another emergent application aiming at addressing this unmet need are adaptive optics-based visual simulators [35]. This approach allows to preoperatively measure and correct the total ocular wavefront, and rapidly simulate differing IOL corrections by incorporating neural processing via direct patient feedback. However, the cataractous lens will cease to be part of the postoperative optical system. While Villegas et al. have shown that lenticular aberrations have an imperceptible effect on IOL profile simulations for a 3-mm pupil size [36], different types of cataract not only induce aberrations or alter the polarity of SA [37], but also cause intraocular scattering which pollutes the cataractous wavefront and reduces contrast sensitivity [38]. Since cataractous straylight can contaminate the preoperatively simulated pseudophakic retinal image, the adjunctive value of adaptive optics simulators remains to be comprehensively evaluated for differing patient demographics. Therefore, clinical decisions guided primarily by corneal aberrations should continue to be endorsed in cataract surgery.

Some limitations of our study are worth addressing. Firstly, it must be emphasized that our results are only valid for healthy cataract eyes and are not applicable for more HOA-dominated optics (i.e., postsurgical or keratoconic eyes). It would be worthwhile to measure pathological eyes in the future because more complex corneal shapes not only require terms above the 4^th^ order, but Zernike polynomials themselves have also shown to fail modelling all the visually significant information in more aberrant corneas [39]. Whether the currently implemented manufacturers’ fitting procedures are still adequate and comparable in these cases, remains to be investigated. Secondly, although this is to our knowledge the first report to fully evaluate the capability of the SS-OCT platform to characterize corneal optical quality, our study was retrospective in nature. However, measurements fulfilled the manufacturers’ quality criteria and our group had already extensive experience with the device [15]. A subsequent prospective randomized repeatability analysis would nevertheless be mandatory, in order to comparatively investigate the impact of eye-tracking, scan pattern density, as well as axial/transversal resolutions on wavefront measurement reliability.

Interestingly, McAlinden et al. have recently found repeated corneal aberrometry from Scheimpflug tomography (Pentacam HR) to be exceedingly precise when numerically adjusting wavefront tilt and misalignment relative to the baseline examination with repeatability/reproducibility limits of < 0.000001 μm up to the 6th Zernike order in healthy eyes [40]. This finding would highlight that irrespective of imaging technology or manufacturer-specific algorithms to define reference plane and unit circle centration, corneal wavefront intra/inter-device variability might merely arise because computations never occur at the exact same location. This issue is inherent to the mutual orthogonality of Zernike terms, which renders them interdependently susceptible to small amounts of tilt and decentration due to ocular misalignments. In healthy eyes, HOAs would thereby be most impacted, given their substantially lower magnitude. As such, statistical differences and inter-device LoAs in our study might simply be reflective of small ocular inter-device misalignments that inevitably altered the characterization of the remnant wavefront error. Since there are still to this day no authoritative guidelines for reporting corneal aberrations, accounting for this aspect may be of considerable interest for future studies [41].

Further improvements to canonize the reporting of corneal aberrations may be also of interest for ray-tracing from corneal imaging because it can obliviate the need for paraxial keratometric assumptions for calculating corneal power, but a caveat of this approach will be the need for stringent data quality requirements. Incorporating all corneal aberrations for customized eye models can increase the degree of sophistication, but also lead to erroneous results due to the introduction of noise [42]. Improving on this aspect could thus potentially facilitate correlations between virtual ray traced aberrations from anterior segment imaging and postoperative whole-eye aberrometry at physiological pupil sizes [43]. Retinal image quality metrics can incorporate the impact of all aberrations on the eyes’ focusing range and enable more comprehensive correlations with subjective visual performance [7, 32, 44, 45]. While proprietary data on exact IOL geometries is ultimately needed to fully employ the nonparaxial regime, this could nevertheless offer the opportunity to assess more thoroughly the predictability of depth of focus extension and its trade-off with contrast sensitivity deterioration.

## Conclusions

Characterizing corneal optical quality from SS-OCT appears to be functionally equivalent to the referential device in regular elderly eyes. Nevertheless, caution should be taken when interpreting clinically relevant higher order aberration parameters for surgical decision-making.

## Data Availability

Available from the corresponding author on reasonable request.

## Abbreviations

ATR: Against-the-rule
HOAs: Higher-order aberrations
IOL: Intraocular lens
LOA: Limits of agreement
OCT: Optical coherence tomography
SD-OCT: Spectral-domain optical coherence tomography
SS-OCT: Swept-source optical coherence tomography
RMS: Root mean square
SA: Spherical aberration
SD: Standard deviation
WTR: With-the-rule
